# Against reflexive recalibration: towards a causal framework for addressing miscalibration

**DOI:** 10.1186/s41512-024-00184-2

**Published:** 2025-02-11

**Authors:** Akshay Swaminathan, Ujwal Srivastava, Lucia Tu, Ivan Lopez, Nigam H. Shah, Andrew J. Vickers

**Affiliations:** 1 Department of Biomedical Data Science, Stanford School of Medicine, Stanford, CA USA; 2 NYU Grossman School of Medicine, New York, NY USA; 3 Nathan S. Kline Institute for Psychiatric Research, Orangeburg, NY USA; 4 Department of Medicine, Stanford School of Medicine, Stanford, CA USA; 5 Memorial Sloan Kettering Cancer Center, New York, NY USA

## Background and significance

Risk prediction models, whether based on traditional statistical approaches or computationally intensive machine learning methods, are increasingly being developed to support decision-making. By estimating individual risks based on multiple predictor variables, prediction models can improve decision-making compared to either clinician judgment or heuristics based on crude risk groups. A critical aspect of risk prediction models is calibration, the extent to which predicted probabilities align with true event probabilities. In a well-calibrated model, close to *x* out of 100 patients given a risk of *x*% will have the event.

Miscalibration can result in harmful care decisions [1]. As an illustration, suppose a myocardial infarction risk model is miscalibrated such that the predicted probabilities are 20-fold lower than true probabilities. Discrimination (such as area-under-the-curve) would be unaffected, but a patient at high risk (e.g., 40%) would be told that they are at low risk (2%) and would forgo beneficial prophylactic therapy.

Here we focus on what to do if a model is found to be miscalibrated upon external validation. One approach would be to immediately update the model by revising model coefficients or modifying the intercept. We refer to this approach as “reflexive recalibration” because it involves mathematically adjusting the model in response to evidence of miscalibration without consideration of underlying causes. In this paper, we discuss some of the dangers of reflexive recalibration and recommend the alternative approach of identifying the causal mechanisms of miscalibration, before deciding on the best course of action.

## Reflexive recalibration

We define reflexive recalibration as any mathematical adjustment to a model made in response to evidence of miscalibration that is done without consideration of the underlying causal mechanisms of the miscalibration.

There are several examples of reflexive recalibration in the literature. The Framingham Coronary Heart Disease (CHD) risk model, for instance, has been evaluated in multiple patient populations and is often reflexively recalibrated. The original Framingham model was developed and internally validated on a predominantly white European population. D’Agostino et al. [2] investigated the generalizability of the Framingham model to a more diverse cohort. Overestimation of risk was found for Japanese American men, Hispanic men, and Native American women. The prediction models were recalibrated by replacing the mean values of the risk factors and incidence rate in the Framingham cohort with their respective values from a non-Framingham cohort. Notably, there was no discussion of why miscalibration was present for these particular groups. Similarly, Hua et al. [3] assessed the validity of the Framingham model in a cohort of Indigenous Australians. They found risk underestimation and reflexively recalibrated models using the same approach as D’Agostino et al. The discussion section of this paper notes the importance of using calibrated models for Indigenous populations but does not discuss why miscalibration was present. Liu et al.’s [4] study of a Chinese population mirrored the approach of Hua et al. and D’Agostino et al.: miscalibration when applied to a different population and modification of the intercept. Changing the intercept is equivalent to adding a coefficient for race, and this approach is typically frowned upon without the presence of a strong causal rationale [5].

The methodological literature often recommends reflexive recalibration when there is evidence of miscalibration. For instance, one group [6] suggested the overarching guideline that “when we find poorly calibrated predictions at validation, algorithm updating should be considered to provide more accurate predictions for new patients from the validation setting.” Furthermore, other workers [7] have explored and compared specific methods for updating a model and concluded that parsimonious model updates (e.g., refitting the intercept) are preferable to more extensive updates (e.g., re-estimating all coefficients). The authors suggest that such recalibration is necessary and sufficient for optimizing a model that is found to be miscalibrated, stating, “If alpha and or beta significantly deviate from the ideal case, there is a need to recalibrate the model.” Some methods go even further than recommending recalibration on evidence of miscalibration: one statistical approach for evaluating prediction models includes a non-parametric recalibration approach hardwired in the methodology [8]; hence, models are automatically recalibrated during the evaluation process without any assessment of the degree of miscalibration.

## Reflexive recalibration ignores the causal pathways leading to miscalibration

Reflexive recalibration undoubtedly solves a problem of scientific publishing: a model that once looked bad now looks good. However, we believe that this approach can obscure problems that impact the value of models when used in clinical practice. Specifically, we think that the first response to miscalibration should be an investigation of causal pathways. Without understanding why a model is miscalibrated, it can be difficult to know whether to use the model, or a recalibrated alternative, in practice. To illustrate this point, imagine that a prediction model for recurrence of cancer after surgery (“Model X”) is created using data from patients in Hospital A. Investigators from Hospital B conduct an external validation study of Model X and find miscalibration. The Hospital B investigators recalibrate Model X, creating a new Model X*. Take the case where the cause of the miscalibration is related to differences in pathology evaluation between the two hospitals. This gives us the following possibilities:

- The pathology approach at Hospital A is more typical; Hospital B is an outlier. In this case, Model X is preferable to Model X* in most populations.
- The pathology approach at Hospital B is more typical; Hospital A is a bit of an outlier. In this case, Model X* is preferable to Model X in most populations.
- The pathology approaches at Hospitals A and B are different but both widely used. In this case, hospitals should select Model X or Model X* according to their approach to pathology.
- There are actually three different common approaches to pathology grading. In this case, the approach would be to create a third Model X2 and decide between Models X, X*, and X2 depending on the pathology approach.
- The pathology approach at Hospital A is more typical and Hospital B is about to switch over to use Hospital A’s approach. In this case, Model X is preferable to Model X*. In other words, the original model should be used in favor of a model recalibrated to a population even in the population used for recalibration.

The key point here is that reflexively recalibrating and using the new model X* would likely lead to outright harm in scenario A and E and suboptimal outcomes in scenarios C and D.

## Understanding “local needs”

Investigators commonly call for models to be recalibrated to “local needs” [6, 9] before deployment in a new population. For instance, one proposal was “a simple method to adjust clinical prediction models to local circumstances” by updating the intercept, which was deemed preferable to developing new models [10] from scratch because it takes advantage of previous predictive information. As an empirical example, Wessler et al. [11] recommend regional recalibration of mortality prediction models in patients with acute heart failure. After assessing the generalizability of existing prediction models derived in North America, they conclude, “performance (specifically calibration) can be improved significantly with simple recalibration procedures, but only when recalibration is performed using region‐specific corrections.” However, there is no consensus on what counts as a “region,” that is, what level of local is appropriate. Should there be, say, one for North America, one for Europe, and one for East Asia? Or should there be different models for different areas of Europe, different countries, or even different regions within countries? It is of note, for instance, that there are likely to be larger differences between patients in London versus North East England than between those in London and Paris. Similarly, there are often larger differences in patient populations in different areas of New York City, than between New York state as a whole and Nebraska.

Perhaps as a result, some studies have recommended going beyond “regional” corrections to recommend hyper-local “site-specific validation” [12]. Although this approach would offer a more accurate picture of model performance at a given site than a more general external validation study, it is currently infeasible without substantial data infrastructure and sufficient patient volume. Take for example a model for predicting the risk of sepsis for patients in intensive care units (ICUs). There are approximately 5000 hospitals in the USA that have an ICU, and in many cases the ICU has fewer than 5 beds [13]. “Site-specific” validation of a sepsis model would be deemed cost and time prohibitive if set up as 5000 separate studies.

As a second example, a study of prediction models for chronic cardiometabolic disease [14] recommended model recalibration “in settings where different disease rates are expected.”

The authors stated that a lower disease incidence rate in the validation cohort than in the development data was the cause of the miscalibration. They reflexively recalibrated by adjusting the intercept, “in line with previous research indicating that simple recalibration techniques seem sufficient for improving performance, especially when discrimination is already adequate in a new setting.” However, it is unclear how to define a “setting.” Again, this could be a unit, a hospital, a city, a region of a country, a whole country, or a continent. The authors themselves explain that incidence rates can be influenced by the specific definition of chronic cardiometabolic disease, smoking prevalence, diet, exercise, and statin use—all factors that vary in unpredictable ways across settings.

## Understanding the causal mechanisms of miscalibration as an alternative approach

We propose that the appropriate response to evidence of miscalibration is not immediate mathematical adjustment of a model, but investigation of the underlying causal mechanisms. We are not the first authors to do so. For instance, Jones et al. have recommended constructing causal diagrams of the data generating process to understand possible mechanisms for miscalibration during model deployment [15]. Similarly, Subbaswamy and Saria propose proactively examining underlying causal mechanisms, as opposed to making “reactive” adjustments, to create transferable models [16]. Moreover, it is clear that this approach fits naturally with more general considerations of good statistical practice. We conduct a study on a sample and say that the results are applicable to future observations drawn from the same population as the study sample. In the specific case of prediction modeling and calibration, we cannot define a population without knowing the causal influences on calibration.

Table 1 gives some examples from the literature where investigators have attempted to determine the root causes of miscalibration. For instance, Ankerst et al. [17] examined influences on models to predict outcomes of prostate biopsies. They found that the coefficient for family history of prostate cancer varied between settings and attributed this to differences in the way that family history was recorded. In research studies, family history is recorded according to protocols that tend to be inclusive (e.g., clinically insignificant cancer diagnosed at advanced age in a second-degree relative); in clinical practice, family history is only recorded if it is remarkable (e.g., aggressive cancer diagnosed at a young age in a close relative). Hence, the coefficient for family history is higher in the latter setting. This insight has clear implications on how to apply models developed using different cohorts.

**Table 1:** Examples of investigating the root causes of miscalibration identified during external validation. These examples were identified through a literature search of PubMed and Google Scholar that included papers published on or before February 4, 2024. We focused on studies that explicitly reported calibration metrics or provided detailed discussions of miscalibration in clinical prediction models. The selection aimed to encompass diverse scenarios, such as biological differences, temporal shifts, and institutional variations, to offer a comprehensive perspective on factors influencing calibration

Dataset shift domain: cause of miscalibration	Specific: variables affected	Real world example	Investigation of miscalibration
Difference in clinical practice	Admission policies, threshold for surgery, medications prescribed, pathology grading	van den Boogaard et al. [18]	A model predicting delirium in ICU patients had poor calibration for participants in a multinational observational study. The model’s overestimation of the risk of developing delirium could be explained by differences in ICU admission policies and treatments, specifically sedation protocols. Varied sedation practices impact the level and duration of sedation, influencing the likelihood and severity of delirium occurrence, therefore affecting the model’s performance.
		Rauh et al. [14]	A model predicting 7-year risk for chronic cardiometabolic diseases had poor calibration for participants in AusDiab, a population-based cross-sectional study. The model overestimated disease rates as it was developed with data from 1989 to 2005 whereas the AusDiab study was conducted from 2004 to 2012—a time period in which there was increased use of antihypertensives and statins.
		Ankerst et al. [17], Vickers [19]	PCPTRC, a model predicting risk of prostate cancer, had poor calibration for patients in both the North American and European cohorts of the Prostate Biopsy Collaborative Group (PCBG). The PCPTRC model’s underestimation of risk may be because of the switch in clinical practice from six-core biopsy procedure to 12 cores. Additionally, there have been changes in how pathologists grade prostate cancer such that there is increased prevalence of high-grade disease in contemporary cohorts.
Difference in human behavior	Diet, exercise, clinician skill	DeFilippis et al. [20]	AHA-ACC-ASCVD, a model for predicting risk of cardiovascular events, had poor calibration in MESA, a multicenter prospective community-based epidemiologic study in a sex-balanced and multiethnic cohort. The model’s overestimation of atherosclerotic cardiovascular disease risk may be explained by differences in salt and trans fat intakes for participants in AHA-ACC-ASCVD’s decades-old development data versus MESA’s modern cohort data.
Difference in data collection techniques	Family history	Ankerst et al. [17]	PCPTRC, a model predicting risk of prostate cancer, had poor calibration for patients in both the North American and European cohorts of the Prostate Biopsy Collaborative Group (PCBG). The PCPTRC model’s underestimation of risk may be because it was built on a screening trial in which family history was a required data element for all participants. In contrast, the PCBG model, which was well-calibrated for both cohorts, was developed using data from clinical records. Clinical records might only include family history of a disease if it was more aggressive (e.g., cancer in a family member might only be noted if it led to death). This difference in family history collection would lead to different odds ratios for family history and thus differences in predicted risk.
Difference in clinical application of the model	Case mix, patient demographics, model setting	Ashburner et al. [21]	CHARGE-AF, a model predicting atrial fibrillation (AF) risk, had poor calibration for participants in a medical record-based study in a tertiary hospital. The model’s underestimation of atrial fibrillation risk can be explained by the fact it was developed in a community setting—with lower incidence of AF—and applied in an academic setting—a population with higher underlying risk of developing AF.
		Das et al. [22]	A model for predicting 30-day mortality in patients with acute myocardial infarction was trained and externally validated on patients enrolled in a randomized controlled trial. When this model was deployed on a community-based cohort, risk was underestimated. This was attributed to differences in patient demographics. Compared to the general population, patients in cardiovascular RCTs tend to be younger, male, undergo more revascularization, and have fewer comorbid conditions.
		Steyerberg et al. [23]	A model predicting indolent prostate cancer that was developed on a cohort in a clinical setting underestimated risks of indolent cancer for a cohort in a screening setting. Patients presenting clinically generally do so for some reason, and therefore have a higher risk of more aggressive disease.
Difference in nomenclature	Definitions, medical coding/billing	Rauh et al. [14]	A model predicting 7-year risk for chronic cardiometabolic diseases had poor calibration for participants in AusDiab, a population-based cross-sectional study. The model overestimated disease rates as its development data defined cardiovascular disease differently from the AusDiab study.
Difference in predictor and outcome relationships	Age, BMI, cholesterol, dementia	Licher et al. [24]	Two models to predict dementia were found to underestimate low risks and overestimate high risks during external validation, even after recalibration by refitting the intercept. The miscalibration was attributed to the original model’s development on a younger cohort compared to the validation cohort, critical because associations between dementia and predictors such as BMI and cholesterol varied by age.

Another example is Ashburner et al. [21], who investigated the use of an atrial fibrillation risk prediction model, CHARGE-AF, in post stroke populations. They found that the original CHARGE-AF model had poor calibration and attributed this to a difference in underlying risk between the development and validation cohorts. CHARGE-AF was developed in a community-based cohort with low baseline AF risk, but it was tested in an academic medical hospital in high-risk stroke patients. The baseline risk tends to be lower in community-based cohorts because they include routine follow-up patients, whereas academic hospitals tend to be referral sites for high-risk patients.

These two examples, along with the others given in Table 1, demonstrate the poverty of calls for local or “site-specific” recalibration. What matters in these examples is not geographic location, and is not specific to each and every site where care is delivered, rather it constitutes generalizable knowledge that can be applied to new settings without the need for further data collection.

Determining the multifactorial causes of miscalibration requires domain expertise and high-quality data, which may not always be available. This presents a challenge: balancing thorough investigation with the practical constraints of time, data availability, and resources. Despite these challenges, we argue that even a partial understanding of the mechanisms driving miscalibration can yield insights that may allow researchers to make informed adjustments that improve model applicability without resorting to a reflexive recalibration.

## Concluding remarks

Miscalibration is commonly found during external validation of a model. We define reflexive recalibration as a mathematical adjustment to a model that is made in response to evidence of miscalibration without consideration of the underlying causal mechanism. We argue that this is a misguided approach and propose that investigators should instead attempt to understand the causal pathways underpinning miscalibration. Doing so can help identify how to best update and implement a model and can result in generalizable knowledge that is transferable to other settings. As such, we are not inherently against recalibration: in our example of a cancer recurrence model, for instance, recalibration would have been of benefit in many scenarios. But such recalibration should only take place after evaluation of causal mechanisms, it should not be reflexive.

## Data Availability

Not applicable.
